# Evaluation of Cellular Changes and Immunohistochemistry Expression of p53 and p16 in the Oral Mucosa Among Saudi Smokers

**DOI:** 10.7759/cureus.55027

**Published:** 2024-02-27

**Authors:** Faris M Elmahdi, Heba E Mostafa, Ali M Eldib, Magda H Youssef, Lama S Alahmadi, Abdullah A Alkurdi, Hend M Hussein

**Affiliations:** 1 Basic Sciences, Al Rayan National College of Medicine, Al-Madinah Al-Monawarah, SAU; 2 Clinical Sciences, Al Rayan National College of Medicine, Al-Madinah Al-Monawarah, SAU; 3 Forensic Medicine and Clinical Toxicology, Faculty of Human Medicine, Zagazig University, Zagazig, EGY; 4 Immunology Division, Zoology, Faculty of Science, Damanhur University, Damanhour, EGY; 5 Medicine, Al Rayan National College of Medicine, Al-Madinah Al-Monawarah, SAU

**Keywords:** saudi smokers, oral mucosa, p16, p53, cellular changes

## Abstract

Background: Smoking is a well-known risk factor for various health problems, including oral cancer. P16 and P53 proteins are involved in cell cycle regulation and proliferation, and their expression levels can provide insights into cellular health.

Objective: This study aims to evaluate the cellular changes and immunohistochemistry expression of p53 and p16 in the oral mucosa among Saudi smokers.

Method: In a cross-sectional study obtained by scraping the buccal mucosa, 1000 samples were collected from 2022 to 2023. All of the study's participants were Saudi citizens of both genders. Seven hundred cigarette smokers and 300 nonsmokers made up the controls, using two sampling techniques: initially purposive and then snowball sampling. The materials were subjected to immunohistochemical analysis for P16 and P53 protein overexpression. The samples were scored based on the percentage of positively stained cells and staining intensity. The data were analyzed using SPSS, and categorical variables were identified as frequencies and percentages using the chi-squared test; a value of (P<0.05) was considered significant.

Result: Cigarette smokers demonstrate significantly higher rates of cytological inflammation, reverse cytological infection, atypia, and binucleated/multinucleated cells compared to nonsmokers, with an overall abnormal result rate of 46% versus 18.7%, respectively (P=0.024). The study found higher P53 and P16 expression among smokers (7.14% and 2.14%, respectively) compared to nonsmokers (0.1% and 0.33%) (P=0.038). No significant differences were observed in P53/P16 expression across age groups (P=0.72) or between male and female participants (P=0.25).

Conclusion: These findings highlight the detrimental effects of smoking on cellular health and reinforce the importance of smoking cessation in reducing the risk of developing cytological abnormalities and associated diseases. These results highlight the association of smoking with increased biomarker expression, emphasizing its relevance in understanding oral health risks.

## Introduction

Smoking tobacco is a major health risk. Worldwide, it is a major contributor to high rates of illness and mortality that may be avoided. Every year, smoking-related diseases claim the lives of five million people worldwide, mostly in underdeveloped nations [1]. Each year, tobacco-related health issues cost the United States $167 billion [2]. Approximately $160 million is spent annually on tobacco purchases in Saudi Arabia [3].

Several studies using different methods, like cytological assessment, DNA damage detection, and histological investigations, have looked at how cells change in the oral mucosa of smokers. These studies have shown that smoking results in a range of alterations, such as impaired cell differentiation, cellular proliferation, and increased epithelial thickness. These alterations might be the consequence of mutations in tumor suppressor and oncogene genes caused by DNA damage from tobacco smoke [4, 5].

P16 is a tumor suppressor protein that controls the cell cycle and stops cells from multiplying [6]. It is also known as cyclin-dependent kinase inhibitor 2A (CDKN2A). The overexpression of P16 has been reported in several studies investigating oral cancer [7]. P53, on the other hand, is a tumor suppressor protein involved in regulating cell cycle progression and preventing the formation of tumors [8]. Increased p53 expression has been associated with various pathological conditions, including oral leukoplakia [9].

Immunohistochemistry (IHC) is a widely used technique for the detection and quantification of protein expression in tissues. It makes proteins in tissue sections visible by using specific antibodies that bind to the target protein and then staining them with a chromogen, which lets you see them under a microscope [10].

The association between smoking and the overexpression of P16 and p53 proteins in various tissues has been investigated in many studies. For example, Sunberg et al. [7] found a significant increase in P16 expression among smokers compared to nonsmokers in oral squamous cell carcinoma. Similarly, Liu et al. [11] reported a positive correlation between smoking and p53 overexpression in oral leukoplakia [12, 13]. The current study investigated the cellular alterations and the immunohistochemical expression of p53 and p16 in the oral mucosa among smokers in Saudi Arabia.

## Materials and methods

For this cross-sectional study, conducted from January to December 2023, 1000 volunteers in good health were chosen at random: 700 cigarette smokers and 300 nonsmokers comprised the controls. The sample size was determined using the Epi Info Software Package Version 7.2 (Centers for Disease Control and Prevention, Atlanta, Georgia) with a confidence level of 95% and a 5% margin of error. Two buccal smears were obtained from each participant (all required safety precautions and sample adequacy measures were followed) [13]. Both smokers and nonsmokers appeared to be in fair health, with ages ranging from 18 to 85 years. All participants were Saudi citizens of both genders, aged above 18 years. Within the study, exclusion criteria included non-Saudi citizens, individuals below the age of 18, and participants who did not provide informed consent.

Sample collection

Buccal Smear

A wooden tongue depressor was used to collect exfoliative cells from the oral mucosa, which includes the tongue's dorsum and both cheeks. The cells were then directly spread on two clean glass slides and were fixed instantly in 95% ethyl alcohol while still moist. Buccal smears were sent to the histopathology lab at the Rayyan College of Medicine in Saudi Arabia for staining and diagnosis.

Papanicolaou’s staining

After being fixed in ethanol, smears were hydrated in a descending series of ethanol solution concentrations (dilutions were performed using distilled water) from 95% to 70% for two minutes each. To detect nuclei, smears were stained for five minutes with Harris hematoxylin. After that, they were rinsed in distilled water to remove any excess stain, differentiated in 0.5% aqueous hydrochloric acid for ten seconds to remove any remaining stain particles, and finally rinsed in distilled water. Following four seconds of dyeing blue in alkaline water, the smears were dehydrated in an ascending series of ethanol concentrations ranging from 70% to 95% twice for two minutes at a time. Smears were incubated for two minutes in Papanicolaou Orange G6 solution, rinsed with 95% ethanol, and then incubated for three minutes with Papanicolaou EA50 staining solution, then checked for cytoplasmic staining. After being dehydrated in 95% pure ethanol, the smears were cleaned in xylene and placed on dibutylphthalate polystyrene xylene (DPX) [13].

Immunocytochemistry

Smears were rinsed with phosphate-buffered saline (PBS) three times for three minutes each. The endogenous peroxidase activity was inhibited by treating each slide with 0.3% hydrogen peroxide in methanol for 15 minutes, followed by three PBS rinses. The use of antibodies (Abs) involved the following: sections were incubated with a primary mouse monoclonal p53 and p16 antibody (Gene Tech Company Limited, Shanghai, China) at a working dilution of 1/100 for 30 minutes at 37 °C; following two washes in PBS, sections were incubated with a secondary antibody, Chem Mate TM EnVision+/HRP (Gene Tech Company Limited), at room temperature for 30 minutes; and finally, sections were washed three times in PBS.

The immunoreactivity was detected using diaminobenzidine (DAB) (Gene Tech Company Limited) diluted 1/100 for 10 minutes as the final chromogen, followed by a three-minute wash in DW. Finally, sections were counterstained for three minutes with hematoxylin, rinsed for five minutes in running tap water, dehydrated in a series of alcoholic solutions, cleaned in xylene, and mounted with DPX. The p53 expression was identified as distinct brown cytoplasmic staining in epithelial cells and nuclei [14].

Cytological evaluation

The Pap-stained smears were examined for abnormalities concerning cytopathology. We searched for indications of keratinization, atypia, inflammation, and infection. Cytological changes were identified by characteristics such as uneven growth and bi- or multinucleation [14].

Statistical analysis

Statistical analysis was conducted using IBM SPSS Statistics for Windows, Version 22 (Released 2013; IBM Corp., Armonk, New York), with a significance level of 0.05. Categorical data were presented using frequencies or proportions, and chi-square tests were employed based on the research questions and type of data.

Ethical consent

Before the specimen was obtained, each participant was requested to complete an ethical consent form in writing. The Al Rayyan Medical Colleges (AMC) Ethical Committee created and approved the informed ethical consent form (Grant no. HA-03-M-122-045).

## Results

Classification of participants

As shown in Table 1, 1000 participants of both genders, encompassing a range of age groups from 18 to 85 years old, participated in the current study. It is noticeable that the ratio of male to female participants (both smokers and nonsmokers) was 1.2:1. Cigarette smokers in the 31-40 age group accounted for the largest percentage (138 out of 700, 19.7%), followed by the 18-20 age group as the second highest (89 out of 700, 12.7%). Among female cigarette smokers, those aged 31-40 years old represented the largest percentage (69 out of 700, 9.9%), while the 18-20 age group followed closely behind (66 out of 700, 9.4%).

**Table 1:** Distribution of smokers and nonsmokers by gender and age group

Male						Female				
Smoker				Nonsmoker		Smoker			Nonsmoker	
Age Group	Number	N (%)		Number	N (%)	Number	N (%)		Number	N (%)
18-20	87	19.7%		40	24.5%	66	25.7%		40	29.2%
21-30	89	20.3%		33	20.3%	54	20.6%		32	23.7%
31-40	138	31.5%		26	16.0%	69	26.3%		20	14.6%
41-50	69	15.8%		30	18.4%	44	16.8%		15	11.0%
51+	55	12.6%		34	20.7%	29	11.1%		30	21.9%

Among smokers, 50 participants (7.14%) tested positive for P53, while 15 participants (2.14%) tested positive for P16. In the nonsmoker's group, only three participants (0.1%) tested positive for P53 and one participant (0.33%) tested positive for P16. The second category in Table 2 pertains to age groups. The results indicate no significant difference (P=0.72) among the age groups. Different age ranges are mentioned, starting from 18-20 and going up to 50+ years. Lastly, the table includes the variable of sex. The P-value is listed as 0.25, suggesting no significant difference between male and female participants. Among males, 42 participants (5.3%) tested positive for P53, while 12 participants (1.6%) tested positive for P16. Among females, eight participants (0.66%) tested positive for P53 and one participant (0.33%) tested positive for P16, as shown in Table 2.

**Table 2:** The prevalence of genetic markers (p53 and p16) across different categories: smoking status, age groups, and sex.

Variable	Category	p53	N (%)	p16	N (%)	Total	N (%)	P-value
Participants	Smokers (n=700)	35	5.0%	15	2.14	50	7.14%	0.041
	Nonsmokers (n=300)	2	0.6%	1	0.33	3	30.10%	
	Total	37	3.7%	16	1.6	53	5.30%	
Age	18–20	0	0.0%	0	0.0%	0	0.0%	0.72
	21–29	0	0.0%	0	0.0%	0	0.0%	
	30–39	8	21.6%	1	6.30%	9	16.90%	
	40–49	12	32.4%	7	43.8	19	35.90%	
	50+	17	46.0%	9	56.3	26	49.1	
Sex	Male	30	81.1%	12	75%	42	79.30%	0.25
	Female	7	18.9%	5	31.30%	12	20.80%	

**Figure 1:**
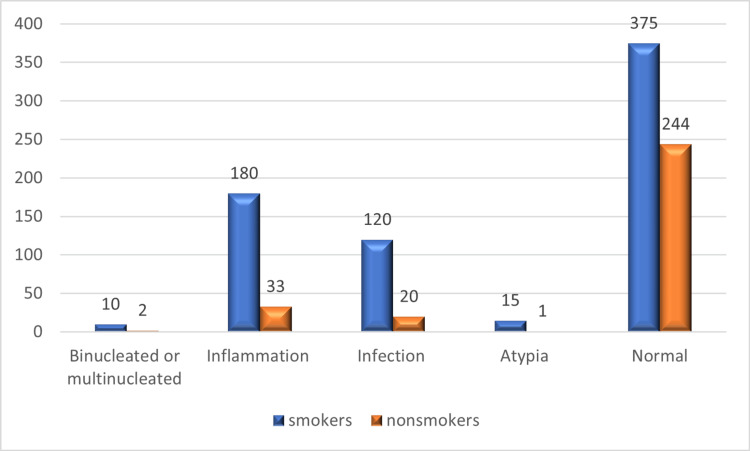
Statistical analysis of cytological changes as shown in smears stained by Papanicolaou’s method.

Four cytopathological changes, each with a distinct proportion, were revealed by interpreting the Papanicolaou staining results: binucleated or multinucleated cells, inflammation, infection, and atypia. As shown in Figure 1, A greater percentage of cigarette smokers 180/700 (25.71%) had cytological inflammation than nonsmokers (33/300). In addition, smokers had greater rates of reverse cytological infection and atypia (120/700 vs. 15/700 respectively) than controls did (20/300 and 1/300 respectively). Binucleated or multinucleated cells were found in cigarette smokers (10/700) when compared to control groups, 2 (2/300). Figure 1 shows that the rate of abnormal results was 18.7% for nonusers and 46% for cigarette smokers (P=0.024). 

**Figure 2:**
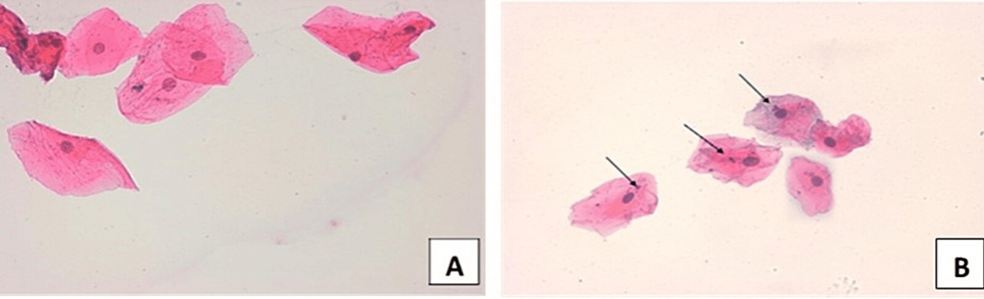
Microphotographs of buccal smears stained using Papanicolaou’s method (x40) and collected from (A) a healthy nonsmoker showing normal epithelial cells and (B) a smoker showing binucleated epithelial cells.

Figure 2 shows buccal smears stained using Papanicolaou’s method of normal control taken from nonsmokers (A) and smokers (B) that reveal binucleated epithelial cells. The number of this type of pathology was detected in 4/100 smoker specimens.

**Figure 3:**
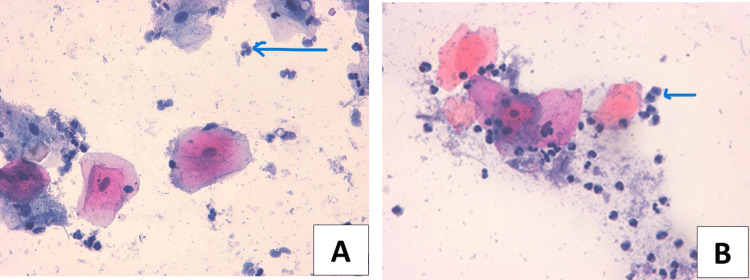
Microphotographs of smear samples from (A) buccal mucosa and (B) tongue of a smoker stained using Papanicolaou’s method (x40) showing inflammation.

The samples taken from different parts of the buccal cavity, buccal mucosa (A), and tongue (B) showed inflammatory cell infiltration, as shown in Figure 3. 

**Figure 4:**
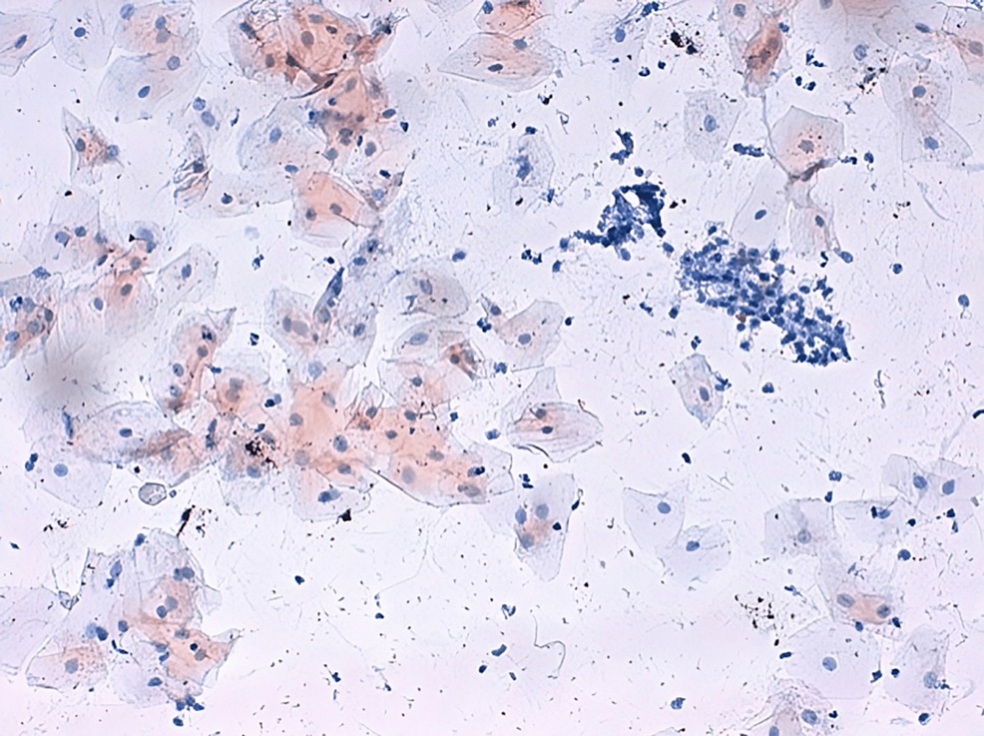
Microphotographs of buccal smear taken from cigarette smokers with immunohistochemical staining, x40 showing expression of p16 in the brown color of the cytoplasm.

Microphotographs of a buccal smear taken from cigarette smokers, with immunohistochemical staining at 40x magnification, show the expression of P16 in the brown color of the cytoplasm, as depicted in Figure 4. 

**Figure 5:**
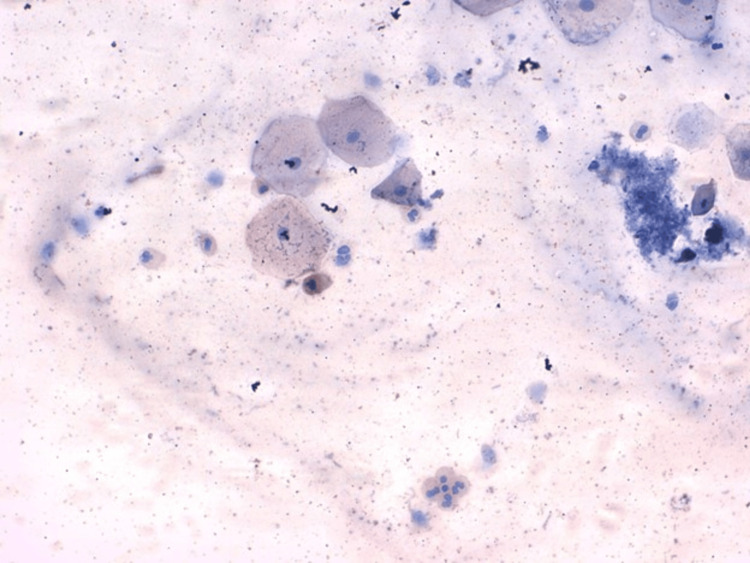
Microphotographs of buccal smear taken from cigarette smokers with immunohistochemical staining, x40 showing expression of p53 in the brown color of the cytoplasm.

## Discussion

Tobacco use, both smoking and chewing, is a risk factor for the development of oral mucosal changes. Tobacco in cigarettes causes an imbalance between antioxidant enzymes in metabolizing and detoxifying carcinogens in tobacco, which may cause changes in the oral epithelium that lead to lesions and dysplasia [15].

In our study, we investigated the classification of participants based on gender and age group, as well as the cytopathological changes observed in cigarette smokers compared to nonsmokers. The results of this study showed that the majority of male cigarette smokers belonged to the 31-40 age group, while female cigarette smokers were most prevalent in the 18-20 age group. The finding of increased smoking prevalence among young adults, particularly in the 18-20 age group for females, aligns with existing literature. Many previous studies have shown that smoking initiation often occurs during adolescence or young adulthood, and early initiation increases the risk of long-term tobacco use and associated health problems [16].

This study showed the results in terms of the relationship between smoking and changes in the oral epithelium. These findings demonstrated a strong link between smoking and the risk of oral epithelial atypical changes, which can lead to precancerous or cancerous changes in the mouth. Similar studies in Africa have shown higher rates, such as the study by Agabeldour et al. from Sudan. Their study involving 80 people who used waterpipes regularly showed that 47.1% had cytomorphologically unusual changes, while none of the controls did. This slight difference from our findings could be attributed to their sample size being smaller than ours [17]. Research indicates that smokers have a higher probability of experiencing alterations in their mouth mucosal epithelia that may result in cancer. Cytologic smear samples were taken from the oral mucosa, the lateral surface of the tongue, and the right side of the mouth floor in 40 waterpipe users and 40 nonusers for a cross-sectional examination. The study found that both smoking and waterpipe use caused measurable changes in the oral mucosa using cytometric analysis. Smoking had a greater impact on cytometric alterations [18]. Another study conducted in Jeddah, Saudi Arabia, revealed that cigarette smoking was the most common form of tobacco use at 65.6%, followed by waterpipe use at 38.1%. When people who smoke were examined in a traditional way, 88.8% of them had soft tissue lesions and a number of oral conditions, including hairy tongues, smoker's melanosis, stomatitis nicotine, frictional keratosis, fissured tongues, gingival or periodontal inflammation, and leukoedema. Concerning (premalignant) lesions were found in only 0.5% of the people. The most common ones were smokeless keratosis (6.3%), leukoplakia (2.3%), erythroplakia (0.7%), oral submucous fibrosis (0.5%), and lichenoid lesions (0.4%). The frequent occurrence of oral mucosal soft tissue lesions (88.8%) is likely due to the irritating effect of tobacco on oral tissues. Yet, in this study, the incidence of cytological changes exceeded that of prior research. This may be due to the presence of cigar smokers and individuals who consume toombak, both of whom can induce abnormal changes in oral epithelial cells within the cohort [19].

This study also relates the presence of cytological inflammation in the oral mucosa to cigarette smoking. Inflammation was found in smokers (23-100) and nonsmokers (11/100). Also, the presence of infection where The buccal smears taken from smokers showed higher incidences of bacterial infections (12%) compared to the control nonsmokers (5%) (with a statistically significant difference). Many studies have shown that cigarette smoking causes a wide range of changes in immunity, leading to inflammation, changing adaptive T-cell-mediated immunity, impaired responses to pathogens, and suppressed anti-tumor immune cell functions. [20]. We examined the expression of p53 in the normal oral mucosa of smokers, as p53 alterations occurring early in oral cavity carcinogenesis are associated with smoking. In our current study, we found a correlation between smoking and p53 expression, which is consistent with findings from several international research projects. The presence of p53 in the mucosa of smokers' samples suggests that smoking may trigger initial mucosal alterations that contribute to the development of oral squamous cell carcinoma (OSCC). Smoking is linked to p53 mutations in OSCC and premalignant lesions. Lazarus et al. suggested that the p53 mutation could be an initial occurrence in the development of oral cavity malignancies [21]. They observed that premalignant lesions in nonsmokers do not exhibit p53 mutations. It is thought that p53 expression above the basal layer in the epithelium is an early sign of oral cancerogenesis and a precursor to carcinoma development, even if there are no other changes in the shape of the tissue [22]. The study showed that smoking is more common among those aged 40 years and above, posing a potential risk to a substantial portion of the population. Therefore, it is crucial to implement strategies to reduce the impact of smoking and to utilize screening programs to effectively manage smoking in the future. A study found that waterpipe use is associated with significant changes in the oral epithelium of Saudi smokers. It is recommended that they participate in frequent screening programs to detect any potential malignant problems [23].

p16 expression was found in three cases of smokers, with 0 expression in nonsmokers; the results are not statistically significant. All the normal cases in this study were found to be negative for the expression of the p16 protein. Contradicting this are the reports on a study in the Indian population by Pandeet et al., which showed 93% of normal tissue to be p16-positive [24]. However, in their study, tissues showing 0% nuclear staining were considered negative, with the range of positivity being 1-10%. However, they do report weak immunoreactivity in normal oral epithelia and normal epithelia adjacent to cancers. The difference in the results may be due to the different criteria for positivity selected in the two studies. Shintianiet al. have also reported a high p16 expression in the studies on normal oral mucosa. Low to undetectable p16 expression has been reported in many normal human tissues analyzed [25]. Gonzalez et al., Yao et al., and Shapiro et al. have reported undetectable levels of p16 in normal human prostate tissue [26-28]. This finding is in conformity with the findings of Lazariuset et al., who found an increase in p16 expression in smokers [29].

Limitations of the study

First, the study design was cross-sectional, which limits our ability to establish causality between smoking and the observed cytopathological changes. Future longitudinal studies are needed to examine whether these cellular changes persist over time and assess their impact on disease development. Second, the study relied on self-reporting of smoking status, which may introduce information bias. Confirmation of smoking status through biochemical markers would strengthen the validity of the results.

## Conclusions

The findings of this study suggest that cigarette smoking is associated with increased expression of p53 and p16, as well as cytological changes indicative of cellular damage and inflammation in the oral cavity. These results highlight the detrimental effects of smoking on oral health and support the need for smoking cessation interventions to reduce the risk of developing oral diseases, including cancer. Further research is warranted to elucidate the molecular mechanisms underlying these associations and to explore potential therapeutic strategies for mitigating the adverse effects of smoking on oral tissues.
